# Expansion of maltose/sucrose related transporters in Ascomycetes and their association with corresponding disaccharide utilization

**DOI:** 10.1016/j.crmicr.2025.100368

**Published:** 2025-03-03

**Authors:** Li Xu, Alessia Manassero, Berend Snel, Ronald P. de Vries, Mao Peng

**Affiliations:** a Fungal Physiology, Westerdijk Fungal Biodiversity Institute, Uppsalalaan 8, 3584 CT, Utrecht, The Netherlands; b Theoretical Biology and Bioinformatics, Biology, Science Faculty, Utrecht University, Padualaan 8, 3584 CH, Utrecht, The Netherlands

**Keywords:** Sugar transporter, Fungi, Evolution, Ascomycota

## Abstract

Sugar transporters (STs) play a crucial role in mediating sugar uptake in fungi and have been increasingly studied due to their important biological roles and industrial potential. In this study, we performed a comprehensive phylogenetic analysis of STs across the fungal kingdom, including species from Ascomycota, Basidiomycota, Mucoromycota and Zoopagomycota. The results revealed a striking diversity of STs among these fungal phyla with respect to their genomic content and predicted sugar specificity. Particularly, we identified a remarkable expansion of maltose/sucrose STs and a strong co-expansion of intracellular α-1,4-glucosidases and invertases in Ascomycota compared to other fungal phyla. In addition, growth profiles support that the utilization of maltose and sucrose across a diverse set of fungi is roughly determined by the presence of both corresponding STs and functionally related hydrolases. This study enhances our understanding of evolutional diversity of fungal STs and provides new insights into metabolic engineering of fungi towards more efficient conversion of plant-derived sugars for relevant industrial applications.

## Highlights

• Phylogenetic analysis reveals diversity in fungal sugar transporters (STs).
• Ascomycota shows notable expansion of maltose/sucrose STs.
• STs and hydrolases related to maltose/sucrose appear to have co-evolved.

## Graphical abstract

**Figure:**
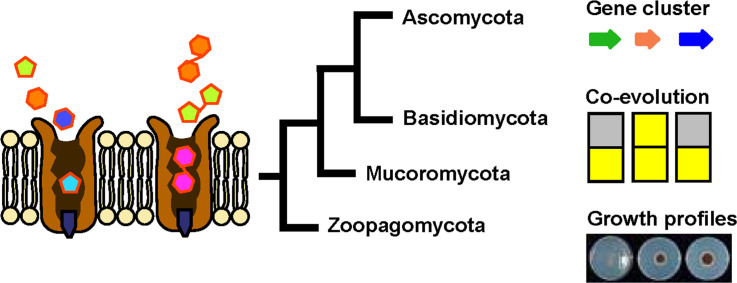

## 1 Introduction

Plant biomass is an important renewable resource for today's bioeconomy. It is mainly composed of cellulose, hemicellulose, pectin, and lignin, as well as storage polysaccharides (e.g., inulin and starch). Fungi are the major degraders of plant biomass in nature, and they have evolved a sophisticated system for plant biomass conversion (PBC) (Culleton et al., 2013). Fungi secrete a variety of Carbohydrate-Active enZymes (CAZymes) to degrade the plant polysaccharides into mono- and small oligosaccharides. These sugars are taken up through specific sugars transporters (STs) and are further metabolized intracellularly.

Due to their important biological function and industrial potential, fungal STs have gained increasing research interest. Several studies have been performed to reveal the functional diversity of STs among commonly studied fungal species. In *Saccharomyces cerevisiae*, >20 STs have been suggested to take part in hexose uptake (Wieczorke et al., 1999). Similar redundancy was found for fungal STs involved in transport of other sugars, such as uronic acid transporters in *Trichoderma reesei* (e.g., GAT1 and GAT2) (Havukainen et al., 2021), and cellodextrin transporters of *Neurospora crassa* (e.g., CDT1 and CDT2) (Znameroski et al., 2014). In addition, comparative genomics has also revealed functional redundancy and evolutionary diversity of STs across different fungal species. Diversity of STs has also been reported for several well-studied filamentous fungi, such as *A. niger, Aspergillus nidulans, T. reesei*, N*. crassa*, and *Penicillium subrubescens* (Nogueira et al., 2020; Xu et al., 2024), indicating the extensive repertoire of STs and their importance in fungal physiology and metabolism.

Besides their major role in mediating sugar transmembrane transport, increasing evidence has shown that genes encoding STs could be co-regulated with other important sugar utilization genes, such as polysaccharide hydrolases and metabolic genes. The mutation or deletion of STs can trigger expressional changes of CAZyme encoding genes in fungi (Cai et al., 2014; dos Reis et al., 2016; Havukainen et al., 2020; Huang et al., 2015; Ivanova et al., 2013; Li et al., 2013; Porciuncula et al., 2013; Zhang et al., 2013). For instance, the deletion of a cellobiose ST gene *cltB* in *A. nidulans* resulted in reduced growth on cellobiose and lower extracellular cellulase activity (dos Reis et al., 2016) whereas in *T. reesei* the deletion of Crt1 (cellulose response transporter 1) resulted in repression of cellulase gene expression (Havukainen et al., 2020; Zhang et al., 2013). In addition, maltose transporters have been found to be tightly co-regulated with corresponding hydrolases by their neighboring regulator within *MAL* loci (gene cluster) in several *Saccharomyces* (Duval et al., 2010; Needleman, 1991) and *Aspergillus* species (Gomi, 2019; Vongsangnak et al., 2009).

Fungal STs are integral to both fungal-plant symbiosis and fungal pathogenicity by facilitating access to plant-produced carbohydrates (Schüßler et al., 2006). For instance, a monosaccharide transporter of a symbiotic glomeromycotan fungus *Geosiphon pyriformis*, GpMST1, is essential for its unique symbiosis with its host cyanobacteria (Schüßler et al., 2006). In *Ustilago maydis*, the maize pathogen, a monosaccharide transporter and sensor Hxt1, is required for virulence (Schuler et al., 2015), while another transporter Srt1, which facilitates the direct uptake of plant-derived sucrose, is necessary for the biotrophic lifestyle, as deletion of *srt1* results in reduced fungal virulence on maize plants (Wahl et al., 2010).

In this study we performed an extensive phylogenetical analysis of STs from PF00083, that belongs to the major facilitator superfamily of a selected set of species across Ascomycota, Basidiomycota, Mucoromycota and Zoopagomycota. PF00083 contains almost all known fungal sugar transporters (Xu et al., 2024), while only few sugar transporters were identified in other PFAM families, e.g., the rhamnose transporter RhtA (Sloothaak et al., 2016), sucrose transporter TvSut (Vargas et al., 2011) and alpha-glucoside transporter Sut1p (Reinders and Ward, 2001). One specific ST clade, the maltose/sucrose STs, showed a clear lineage-specific expansion in Ascomycetes, and we investigated their co-evolution pattern with corresponding hydrolases, and compared the phylogenetic profiles to fungal growth profiles on corresponding sugars. Our study provides a deeper and more systematical understanding of evolutional diversity of fungal STs, especially the maltose/sucrose utilization system, which could facilitate future genetic engineering of fungi towards more efficient conversion of plant-derived sugars for relevant industrial applications.

## 2 Materials and methods

### 2.1 Identification of ST encoding genes across fungi kingdom

The proteome sequences of 45 fungi (including 26 Ascomycota, 15 Basidiomycota, two Mucoromycota and two Zoopagomycota) were downloaded from the JGI MycoCosm database (Grigoriev et al., 2014) (Table S1). All proteomes were filtered by BUSCO (Simão et al., 2015) using fungi_odb10 database with >90 % completeness. The ST domain (PF00083) profile obtained from the PFAM database was used to search against the sequences of the downloaded proteomes and collected known STs using the “hmmsearch” of the HMMER tool (Eddy, 1998). The lowest score (233.2) observed among the “hmmsearch” results of all the known STs was set as a cutoff.

### 2.2 Identification of α-1,4-glucosidase, glucoamylase and invertase encoding genes and gene clusters

The amino acid sequences of four glycoside hydrolases (GHs) families involved in hydrolyzing α-1,4-glucosidic bond of maltose (GH13, GH31, GH15) or β-2,1-glycosidic bond of sucrose (GH32) were collected from JGI MycoCosm (Grigoriev et al., 2014), except for the GHs of *Penicillium brasilianum* that were selected based on a previous study (Lenz et al., 2022). Enzyme specificity of selected GHs was assigned through incorporating characterized fungal enzymes in the phylogenetic tree based on the assumption that the genes of the same clade share a similar function (Eisen and Wu, 2002). The secretion signal peptides were predicted by three tools (SignalP 6.0 (Teufel et al., 2022), Phobius (Käll et al., 2007), and TOPCONS (Tsirigos et al., 2015)). Only the signal peptides that are supported by at least two tools were accepted in this study.

The prediction of the *MAL* gene cluster in Ascomycota was based on Blast search of the well-studied *Aspergillus oryzae MAL* clusters (Hasegawa et al., 2010). The corresponding transporters and α-1,4-glucosidases were selected based on Blast p-value 〈 1.0E-75 and percent identity 〉 50 %. The neighboring genes next to α-1,4-glucosidases or transporters that encode a transcription factor were assigned as putative maltose regulator. The Blast analysis was performed on JGI MycoCosm database. The obtained gene clusters were visualized by clinker (Gilchrist and Chooi, 2021).

### 2.3 Sequence alignment and phylogenetic analysis

Protein sequences of predicted STs were aligned with MAFFT (Katoh and Standley, 2013). The positions that contained >20 % gaps were removed from the alignment using trimAl (Capella-Gutiérrez et al., 2009). The phylogenetic analysis was performed with maximum likelihood method using IQ-TREE 2 (Minh et al., 2020) with 1000 UFBoot2 bootstrapping and MFP option (using ModelFinder to determine the best-fit model) (Kalyaanamoorthy et al., 2017). We further predicted sugar specificity of the STs by incorporating characterized STs into the phylogenetic tree, based on the assumption that genes with high amino acid sequence homology tend to share similar functions (Eisen and Wu, 2002).

For the species tree, whole proteomes of the selected Ascomycota, Basidiomycota, Mucoromycota and Zoopagomycota species were compared and clustered to identify single-copy genes using OrthoFinder (Emms and Kelly, 2019). The protein sequences of single-copy genes were aligned and concatenated to infer an approximately-maximum-likelihood phylogenetic tree using FastTree 2 (Price et al., 2010). The resulting tree shares a similar topology as the tree of fungi on Mycocosm (Grigoriev et al., 2014), and was visualized with iTOL (Letunic and Bork, 2016).

### 2.4 Estimation of divergence time, gene family expansion and contractions of maltose/sucrose STs

The species tree was used to estimate divergence times using r8 s (v1.81) (Sanderson, 2003). Two calibration points obtained from TimeTree (Kumar et al., 2022) were applied to the dating analysis, including 642 and 769 million years ago (MYA) for the divergence time for *A. niger* - *Dichomitus squalens* and *A. niger* - *Mucor lusitanicus*, respectively.

The species tree with estimated divergence time together with the gene counts of each gene othologs family (including the maltose/sucrose ST) per species were applied to investigate the expansion and contraction of each gene family by CAFE 4 (De Bie et al., 2006) with lambda 0.001.

### 2.5 Growth profiling

For growth phenotype analyses, strains were grown on minimal medium (MM) (de Vries et al., 2004) on 1.5 % (w/v) agar plates containing the following (per liter): 6.0 g of NaNO_3_, 1.5 g of KH_2_PO_4_, 0.5 g of KCl, 0.5 g of MgSO_4_, 200 μl of trace elements (10 g of EDTA/liter, 4.4 g of ZnSO_4_ · 7H_2_O/liter, 1.01 g of MnCl_2_ · 4H_2_O/liter, 0.32 g of CoCl_2_ · 6H_2_O/liter, 0.315 g of CuSO_4_ · 5H_2_O/liter, 0.22 g of (NH_4_)6Mo_7_O_24_ · 4H_2_O/liter, 1.47 g of CaCl_2_ · 2H_2_O/liter, and 1.0 g of FeSO_4_ · 7H_2_O/liter, supplemented with one of three carbon sources, including 25 mM d-glucose, 25 mM maltose, 25 mM sucrose. Growth was performed at 30 °C for Aspergilli and 25 °C for the other species. Fungal growth on media with no carbon source and glucose were used as a control. If growth on a specific carbon source is the same as on no carbon source, it is considered as no growth.

## 3 Results

### 3.1 STs are selectively expanded in Ascomycete compared to other fungal phyla

**Fig. 1:**
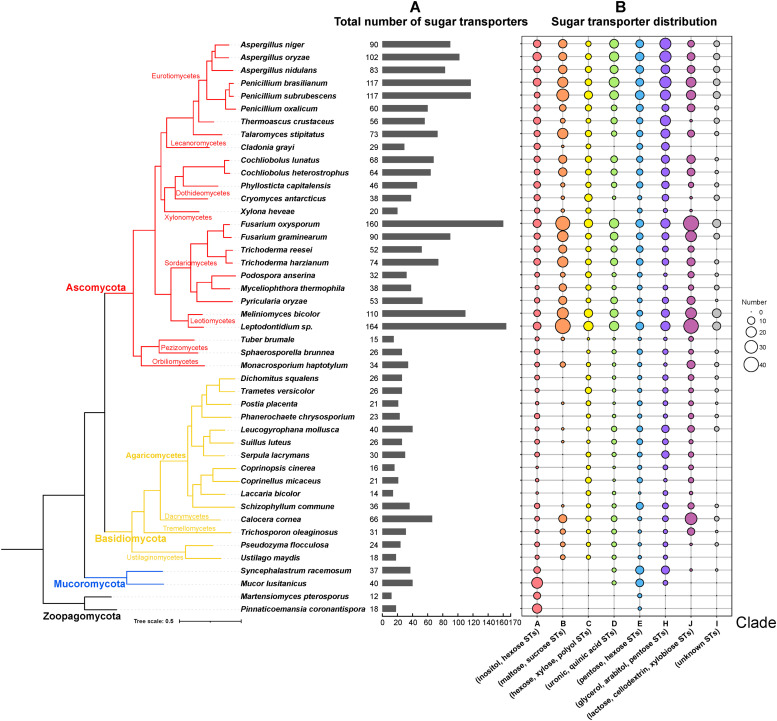
The distribution of predicted sugar transporters (STs) in the selected species across the whole fungi kingdom. (A) the predicted total STs in each species. (B) the number of STs identified in each ST clade for each species. The species tree was based on the complete set of orthologous single-copy genes of the studied species, and the ST clades were defined through phylogenetic analysis of all predicted STs (see details in Method part and Fig. S1).

The total number of predicted STs showed strong diversity across the fungal kingdom based on the selected fungal species (Fig. 1A). In general, Ascomycete genomes contain more STs (average 90) than Basidiomycete genomes (average 26). Genomes of two early-diverging classes Mucoromycetes and Zoopagomycetes comprise on average 38 and 15 STs, respectively. *Leptodontidium sp*. and *Martensiomyces pterosporus* contain the largest and smallest numbers of STs in their genome (164 and 12, respectively). In the tested Ascomycetes, the total number of predicted STs varies significantly between and within the genera (15 to 164). In Eurotiomycetes, the three evaluated species of *Aspergillus* exhibit a similar number, while *P. oxalicum* has around half the number of the other two analyzed *Penicillium* species. Similar variation was observed for the Dothideomycetes and Sordariomycetes. For instance, the total number of STs of two *Fusarium* species are higher than those of other species. In two less well-studied Ascomycota genera, Pezizomycetes and Orbiliomycetes, a relatively small number of STs (15–34) were identified. Basidiomycete species have relatively similar numbers of STs, ranging from 18 to 40, except for the low number (14) of STs identified in *Laccaria bicolor* (a mycorrhizal species) and high number (66) in *Calocera cornea*.

**Fig. 2:**
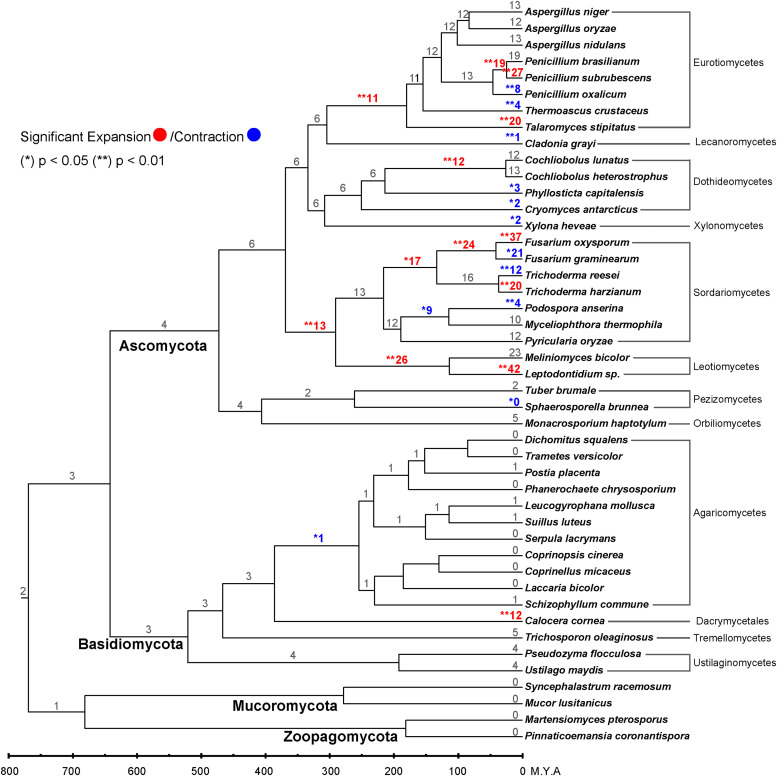
Maltose/sucrose STs expansions and contractions in 45 fungi. Significant expansions and contractions together with the gene numbers were indicated with red and blue, respectively.

Compared to a previous study (Xu et al., 2024), we identified eight major ST clades, in which seven clades are similar between these two studies, while the current clade E combines three previous clades (previous clade E, F, G) due to topology change of the tree with more STs from diverse species were integrated for analysis than the previous study (Fig. S1). We further predicted sugar specificity of the STs by incorporating characterized STs into the phylogenetic tree, based on the assumption that genes within the same clade tend to share similar functions (Eisen and Wu, 2002). The predicted specificity of STs includes different monosaccharides and/or polyols (clade A, C, E, and H), disaccharides (clade B), uronic acids (clade D), and short oligosaccharides (clade J) (Xu et al., 2024). The distribution of ST clades showed remarkable variation across different fungal phyla (Fig. 1B), which indicates a strong functional diversification of STs during fungal evolution. In early-diverging fungal lineages, the two selected Zoopagomycetes fungi only harbor STs from clade A and clade E that are mainly involved in transport of inositol, hexose and pentose, while the two Mucoromycetes also have STs from two additional clades (i.e., D and H) for uptake of uronic acids and sugar alcohols. Most Dikarya fungi have STs from six of the eight clades. In general, Ascomycete species have a relative higher number of STs than Basidiomycetes in each clade, especially in Clade B, D, H and J. One exception is *Calocera cornea* that has a larger number (66) and more diverse of STs than most of other Basidiomycetes. Notably, clade B, containing maltose & sucrose STs, is significantly expanded in most of the studied Ascomycetes, but was reduced or not significant changed in most of the Basidiomycetes, except the *C. cornea* (Fig. 2). This remarkable expansion suggests that several Ascomycetes have developed maltose and sucrose import to increase their competitiveness by importing disaccharides composed of preferred monosaccharides instead of producing monosaccharides in the presence of environmental competitors.

### 3.2 *MAL* gene clusters are more conserved in Eurotiomycetes

**Fig. 3:**
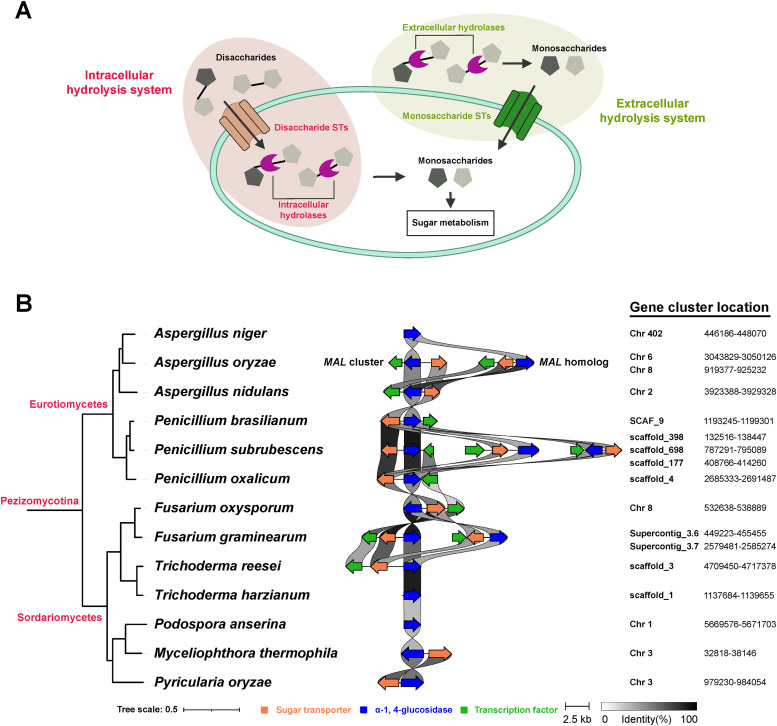
Fungal maltose/sucrose utilization systems. (A) Two different fungal maltose/sucrose utilization approaches. **(**B) Presence of *MAL* cluster of *Aspergillus oryzae* in a diverse set of Eurotiomycetes and Sordariomycetes. Homologous gene clusters searches were only performed in the selected Ascomycetes and were not detected in the non-Ascomycetes of this study. The identity threshold for links is 30 %. The corresponding protein IDs are listed in Table S2.

Fungi have two approaches to utilize maltose or sucrose (Fig. 3A). In the first approach, referred to as intracellular hydrolysis system, the disaccharides enter the cell through the maltose/sucrose transporters and are then intracellularly hydrolyzed to monosaccharides, while in the extracellular hydrolysis system the disaccharides are first extracellularly hydrolyzed into monosaccharides that enter the cell through the universally present hexose STs. Given the unique lineage-specific expansion of maltose/sucrose STs in common studied species of Ascomycota and their strong functional association with intracellular hydrolases, we investigated whether there is a clear co-evolution pattern between these STs and corresponding hydrolases at both gene cluster level and overall gene content level and assessed the possible correlation between corresponding phylogenetic profiles of STs and hydrolases and fungal growth profiles on specific sugars.

Some of the genes involved in maltose utilization have been found to be closely clustered together in fungal genomes, such as *amyR-agdA-amyA* gene cluster in *Aspergillus oryzae* (Gomi, 2019; Gomi et al., 2000) and *MAL* cluster in several *Saccharomyces* (Duval et al., 2010; Needleman, 1991) and *Aspergillus* species (Gomi, 2019; Hasegawa et al., 2010; Vongsangnak et al., 2009). In the *MAL* cluster, a maltose transporter encoding gene is co-regulated with an α-1,4-glucosidase encoding gene under the control of MalR. A search of homologous clusters of a functional and a non-functional *MAL* cluster of *A. oryzae* (Hasegawa et al., 2010), was performed in all selected species. The *MAL* clusters were identified in most Eurotiomycetes (except *A. niger*) and three Sordariomycetes (two *Fusarium* species and *T. reesei*) (Fig. 3B). In line with previous studies (Gomi, 2019), the *MAL* cluster of *A. nidulans* showed higher identity to the non-functional cluster (*MAL* homolog) of *A. oryzae. P. subrubescens* has three copies of the *MAL* cluster, while *Myceliophthora thermophila* and *Pyricularia oryzae* possess an incomplete *MAL* gene cluster lacking the regulator. Interestingly, all the α-1,4-glucosidases identified in these *MAL* gene clusters encode intracellular GH13 enzymes, which suggests they work in tandem with the neighboring maltose STs.

### 3.3 Concurrent expansion or absence of maltose/sucrose transporters and corresponding intracellular hydrolases

**Fig. 4:**
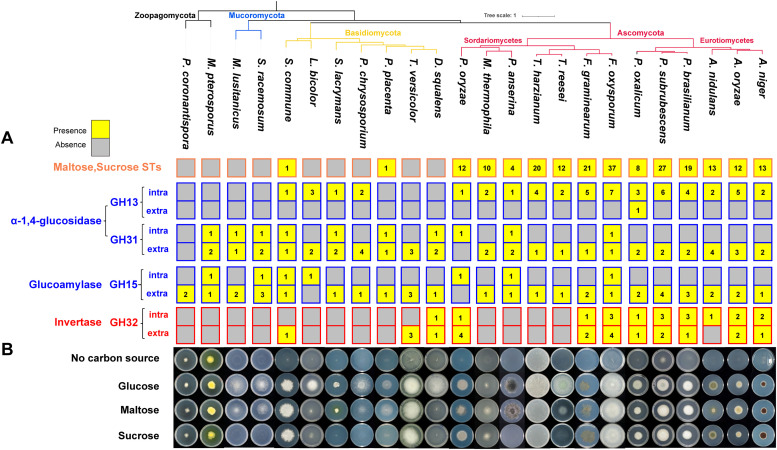
The presence of STs and hydrolases related to maltose/sucrose utilization roughly determines fungal growth on the corresponding disaccharides. (A) The presence and absence of maltose/sucrose STs and corresponding hydrolases. The presence and absence of a gene is highlighted in yellow and grey, respectively. If gene is present, the corresponding copies numbers were shown. (B) Fungal growth profiling on maltose and sucrose, and two controls (glucose and no carbon source).

GH13 (Fig. S2) and GH31 (Fig. S3) α-1,4-glucosidases (Auiewiriyanukul et al., 2018; Nakamura et al., 1997) and GH15 glucoamylases (Fig. S4) are involved in maltose hydrolysis (Nunberg et al., 1984), while GH 32 invertases (Fig. S5) (Xu et al., 2014) are involved in sucrose hydrolysis. The functional association between maltose/sucrose STs and corresponding intracellular hydrolases correlates with a strong concurrent presence/absence profile across the selected commonly-studied fungi (Fig. 4A). Both maltose/sucrose STs and GH13 intracellular α-1,4-glucosidases have expanded in the selected Eurotiomycetes and Sordariomycetes of this study, but are absent in two early branching fungal phyla (Mucoromycetes and Zoopagomycetes). In contrast, no clear correlation was observed in the tested Agaricomycetes, as two of these Basidiomycete species possess maltose/sucrose STs, while the intracellular GH13 enzymes were identified in four species (Fig. 4A). In addition, no clear correlation was identified between GH31 α-1,4-glucosidase and GH15 glucoamylases with maltose/sucrose STs. The extracellular GH31 and GH15 enzymes were identified in nearly all the tested species, while the intracellular GH31 and GH15 were identified in a scattered pattern in the non-Eurotiomycetes (Fig. 4A).

The intracellular invertase (GH32) showed a clear co-occurrence pattern with the maltose/sucrose STs in the Eurotiomycetes and two early branching fungal phyla, but not in several Sordariomycetes and tested Agaricomycetes. Most species have either no invertases or both the intracellular and extracellular invertases, with the exception of *A. nidulans* that only possess one intracellular invertase and two Agaricomycetes species (*Schizophyllum commune* and *Trametes versicolor*) that have one and three extracellular invertases, respectively.

### 3.4 The presence of STs and hydrolases related to maltose/sucrose utilization partially contributes to fungal growth on these disaccharides

The phylogenetic profiles of maltose/sucrose STs and corresponding hydrolases mentioned above suggest that most of the studied species can use maltose as a carbon source, while only the Eurotiomycetes, and a few Sordariomycetes and Agaricomycetes showed obvious growth on sucrose (Fig. 4B). More specifically, most Eurotiomycetes, Sordariomycetes and two Agaricomycetes (*S. commune* and *Postia placenta*) have both the intracellular (mainly GH13 and disaccharide STs) and extracellular (GH31, GH15 and universally present hexose STs) maltose hydrolysis system, while the other species appear to rely mainly on extracellular hydrolases from GH31 and GH15 due to their lack of maltose/sucrose STs. In contrast, the complete set of sucrose utilization genes (both intracellular and extracellular) have only been identified in Eurotiomycetes and three Sordariomycetes. Only three Agaricomycetes possess extracellular invertases, and no Agaricomycete has a combined maltose/sucrose ST and intracellular invertase system for sucrose. To validate the predicted diversity of sucrose/maltose utilization approaches among different fungi, we comparatively analyzed the growth profile of these fungi on maltose and sucrose (Fig. 4B).

In line with the common presence of maltose utilization genes in all studied species, we observed improved growth of almost all the tested fungi on maltose compared to no carbon source, except for *P. coronantispora* that showed similar growth on all of the tested conditions. Most species that possess both intracellular and extracellular maltose hydrolysis systems showed relatively better growth on maltose compared to glucose, except for *A. nidulans, P. oxalicum* and two *Trichoderma* species (Fig. 4B). In contrast, poor growth on maltose was observed for *L. bicolor* and *D. squalens* that lack maltose/sucrose STs but have extracellular α-1,4-glucosidases, suggesting that expression of these genes is not induced by maltose.

Similarly, we observed good growth of Eurotiomycetes and three Sordariomycetes (two *Fusarium* and *P. oryzae*) on sucrose, which could be explained by their complete gene sets for both intracellular and extracellular sucrose utilization. Even though *A. nidulans* only has intracellular invertases, and two Agaricomycetes (*S. commune* and *T. versicolor*) only have extracellular hydrolases, these species also showed considerable growth on sucrose. Most of the tested Sordariomycetes, selected Agaricomycetes, Mucoromycetes, and Zoopagomycetes that lack invertase, showed no or poor growth on sucrose, with the exception of M*. thermophila* that grows on sucrose in the absence of both intra- and extracellular invertase (Fig. 4B). Similar as for maltose, poor growth was observed for *D. squalens* despite possessing an extracellular invertase encoding gene.

## 4 Discussion

STs play a crucial role in the acquisition of carbon by many fungi but have so far received relatively little attention. Previous sugar transport studies mainly focused on detailed functional characterization of specific STs in a limited set of species or performed overall gene content comparison across a diverse set of fungal species. In contrast, in this study we performed phylogenetic analysis of STs and their functionally related CAZymes across the fungal kingdom, and integrated the resulting phylogenetical profiles with fungal growth phenotypes on specific sugars. The remarkable diversity of the total set of STs identified in this study supports the broad role and fast evolution of STs in facilitating the development of various osmotrophic approaches in fungi to adapt to their specific biotopes (Merényi et al., 2023). For example, the plant pathogen *F. oxysporum* shows a large expansion of STs, whereas the mycorrhizae *L. bicolor*, has the fewest STs in Dikarya.

Phylogenetic analysis of the major sugar transporter family (PF00083) reveals a detailed picture of STs distribution and evolutionary history in the studied species. In addition to maltose/sucrose STs (clade B), which expanded specifically in most of the studied Ascomycetes, other clades also showed unique evolution patterns. For instance, clade C with a predicted role in transporting hexose, xylose and polyols was exclusively present in Dikarya. In addition to the prominent expansion of clade B in Ascomycetes, lower levels of expansion were also observed for these fungi in clade D and J with predicted functions in transporting uronic acids and small oligosaccharides, respectively. To adapt to complex and diverse sugar composition in plant biomass, the evolution of osmotrophy in fungi correlates with gene expansion and diversification for transporters and CAZymes (Merényi et al., 2023). Especially, the co-expansion of maltose/sucrose STs and corresponding hydrolases correlated with good growth on these disaccharides in saprobes and plant pathogens in this study. Previous studies have demonstrated GH32 gene expansion in fungal plant pathogens and saprobic fungi (Parrent et al., 2009; Van der Nest et al., 2015), and suggested an important role in mediating the use of plant-derived sucrose. Further investigation into biological activities of STs across a wide range of species is still needed to deepen our understanding of the evolutionary diversity of STs. For example, a previous study suggested that pectin hydrolase family GH28 showed lineage-specific expansions in necrotrophic fungal pathogens (Sprockett et al., 2011). In our data, we observed a correlation between the number of GH28 pectinases and clade D (uronic acids STs) transporters in our studied fungi (Table S8), suggesting that necrotrophic fungi would likely also have increased numbers of clade D transporters. Different from previous studies focusing on degrading enzymes as fungal pathogenicity factors, our study suggests that the remarkable expansion of sucrose and uronic acid STs in necrotrophic fungi could also contribute to their invasion of plant host. Design of specific ST inhibitors to interfere with sucrose and uronic acid utilization of plant pathogens could guide the development of novel fungicides.

Fungal growth on specific carbon sources is a complex interplay of various factors, including the availability of transporters, the presence of enzymes capable of hydrolyzing complex sugars, related metabolic pathways, and the regulation of gene expression. For example, the two studied Zoopagomycetes have no d-galacturonic acid STs (clade D), no GH28 enzymes that could hydrolyze pectin, nor the related d-galacturonic acid metabolic genes (Table S8). Our results support that the overall presence/absence of a complete gene set for intracellular/extracellular maltose/sucrose utilization correlates well with the growth profiles on these disaccharides. However, we also observed some exceptions that suggest that there may be alternative, unidentified STs (e.g., ABC transporters or members of other MFS subfamilies) and hydrolases that could be involved in fungal maltose/sucrose utilization. For instance, the absence of maltose/sucrose STs in *T. versicolor* does not correlate with the good growth of this fungus on sucrose, which suggests that alternative STs could mediate the transmembrane transport of sucrose in this species. In addition, M*. thermophila* lacks invertase, but shows considerable growth on sucrose (Fig. 4), indicating that this fungus has developed novel strategies for sucrose utilization. Invertase encoding genes are also absent in two *Myceliophthora heterothallica* genomes available on Mycocosm (Grigoriev et al., 2014) supporting the true absence of these genes in these species. Discovery of these alternative, unidentified STs and hydrolases would provide novel insights into design the fungal cell factories for more efficient utilization of corresponding oligosaccharides, and accelerating the valorization of plant biomass.

## 5 Conclusion

In this study, the sugar transportome of selected fungi across the fungal kingdom were comparatively analyzed. The remarkable diversity of STs revealed in this study supports their crucial role in facilitating fungal adaption to different biotopes. In addition, through combinatorial analysis of the phylogenetical profiles of sucrose/maltose STs and corresponding hydrolases and fungal growth profiles, we revealed the lineage-specific co-evolution of sucrose/maltose utilization STs and hydrolases. Given the huge industrial potential of fungal STs, the new insights derived from this study could enhance our understanding of STs in fungal physiology and pathogenicity, and guide further metabolic engineering of fungi towards more efficient valorization of plant-derived sugars for relevant industrial applications.

## CRediT authorship contribution statement

**Li Xu:** Formal analysis, Visualization, Writing – original draft. **Alessia Manassero:** Investigation. **Berend Snel:** Conceptualization, Writing – review & editing. **Ronald P. de Vries:** Conceptualization, Supervision, Writing – review & editing. **Mao Peng:** Conceptualization, Supervision, Writing – review & editing.

## Declaration of competing interest

The authors declare the following financial interests/personal relationships which may be considered as potential competing interests:

Li Xu reports financial support was provided by Chinese Scholarship Council. If there are other authors, they declare that they have no known competing financial interests or personal relationships that could have appeared to influence the work reported in this paper.

## Data Availability

All data is available publicly through mycocosm (https://mycocosm.jgi.doe.gov/mycocosm/home)

## Appendix Supplementary materials

Image, application 1

Image, application 2

Image, application 3

Image, application 4

Image, application 5

Image, application 6

Image, application 7

Image, application 8

Image, application 9

Image, application 10

Image, application 11

Image, application 12

Image, application 13
