# Supplementary material for: Expansion of maltose/sucrose related transporters in Ascomycetes and their association with corresponding disaccharide utilization
Source: Curr Res Microb Sci. 2025 Mar 3;8:100368. doi: 10.1016/j.crmicr.2025.100368 (PMC11930586; doi:10.1016/j.crmicr.2025.100368)

Supplementary figure S1. Phylogenetic tree of predicted STs from the 45 fungi of this study and previously experimentally characterized fungal STs. Branches with bootstrap values > 60 % are indicated at the nodes with grey circles. Different clades derived from phylogenetic analysis are highlighted in different colors. STs from different class are marked with different colors. The details of protein sequences and annotation were listed in Table S3.

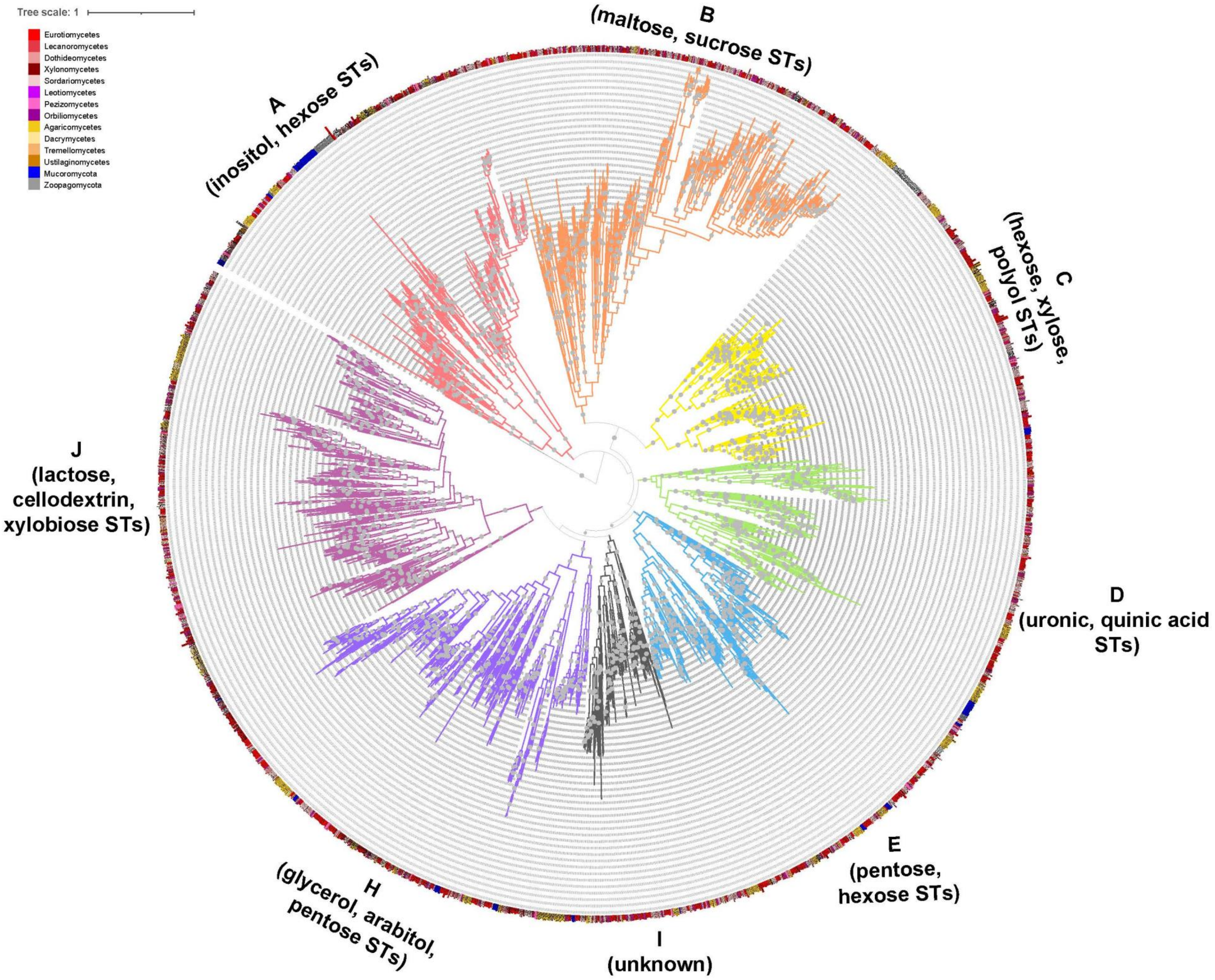

Supplement: Supplementary file 1 [file mmc1.pdf]
