# Supplementary material for: Expansion of maltose/sucrose related transporters in Ascomycetes and their association with corresponding disaccharide utilization
Source: Curr Res Microb Sci. 2025 Mar 3;8:100368. doi: 10.1016/j.crmicr.2025.100368 (PMC11930586; doi:10.1016/j.crmicr.2025.100368)

Supplementary Fig. S2. Phylogenetic classification of GH13 from 24 selected fungi in this study and previously experimentally characterized fungal GH13 genes related to maltose hydrolysis. N and Y were used to indicate the absence and presence of a signal peptide, respectively. GenBank or Unipot accession numbers are shown for experimentally characterized enzymes, while the JGI protein IDs were shown for other enzymes. Only the clade containing the characterized fungal α-1,4-glucosidase is highlight in orange. The details of protein sequences and annotation are listed in Supplementary Table S4.

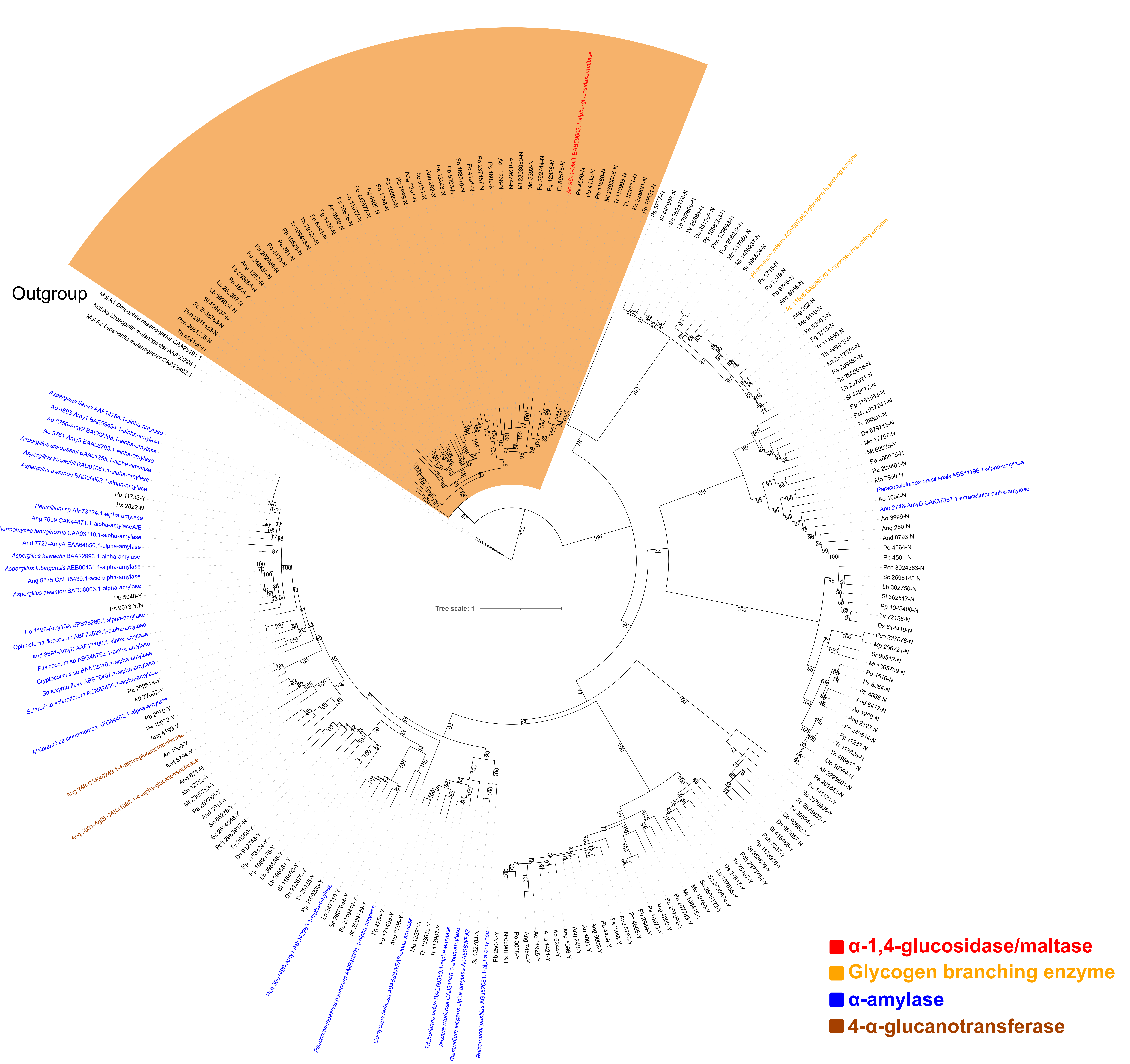

Supplement: Supplementary file 2 [file mmc2.pdf]
