# Supplementary material for: Expansion of maltose/sucrose related transporters in Ascomycetes and their association with corresponding disaccharide utilization
Source: Curr Res Microb Sci. 2025 Mar 3;8:100368. doi: 10.1016/j.crmicr.2025.100368 (PMC11930586; doi:10.1016/j.crmicr.2025.100368)

Supplementary Fig. S3. Phylogenetic classification of GH31 from 24 selected fungi in this study and previously experimentally characterized fungal GH31 genes related to maltose hydrolysis. N and Y were used to indicate the absence and presence of a signal peptide, respectively. GenBank accession numbers were shown for experimentally characterized enzymes, while the JGI protein IDs were shown for other enzymes. Only the clade containing the characterized fungal α-1,4-glucosidase is highlight in orange. The details of protein sequences and annotation are listed in Supplementary Table S5.

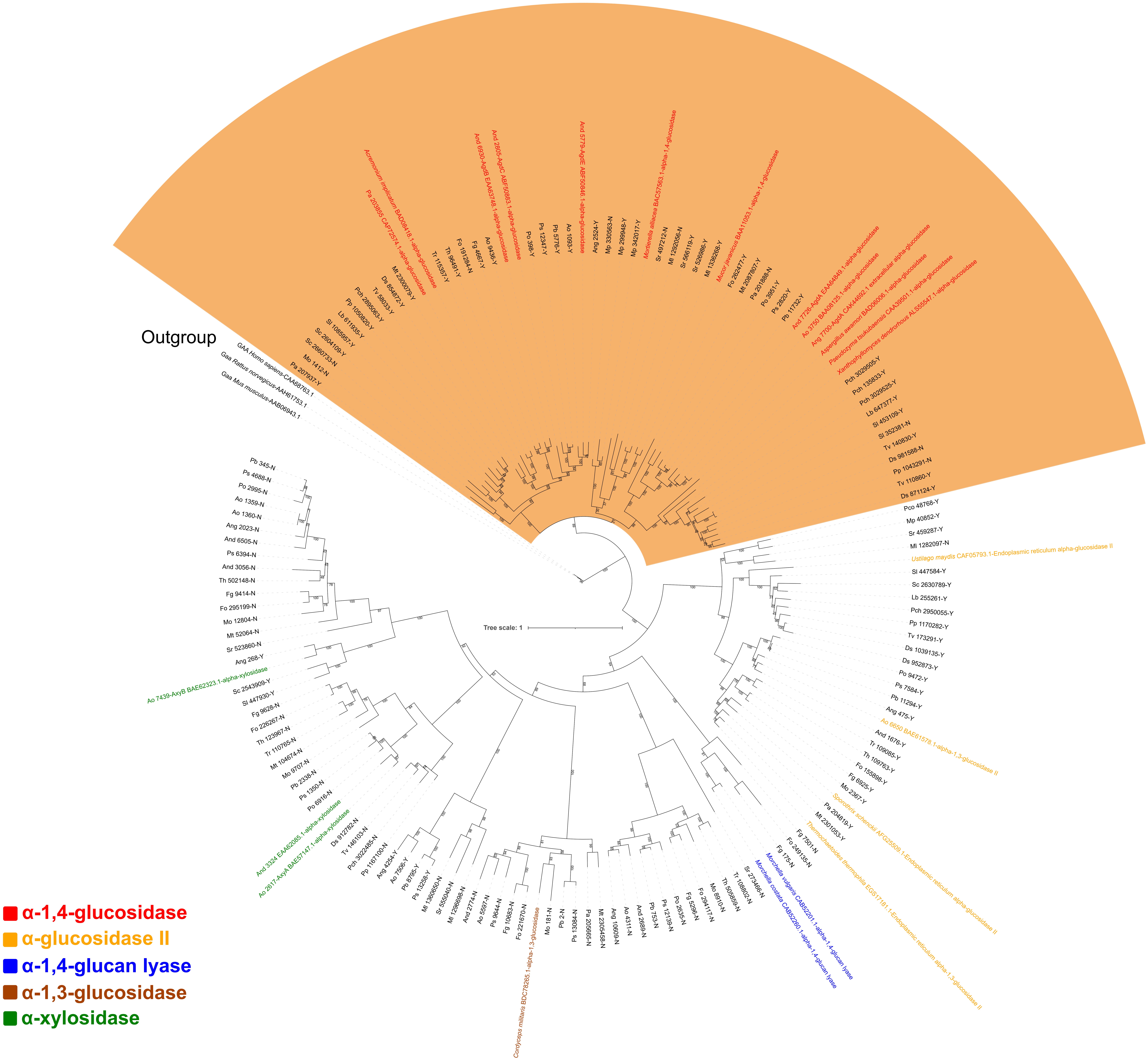

Supplement: Supplementary file 3 [file mmc3.pdf]
