# Supplementary figures and images for: Expansion of maltose/sucrose related transporters in Ascomycetes and their association with corresponding disaccharide utilization
Source: Curr Res Microb Sci. 2025 Mar 3;8:100368. doi: 10.1016/j.crmicr.2025.100368 (PMC11930586; doi:10.1016/j.crmicr.2025.100368)

# outgroup

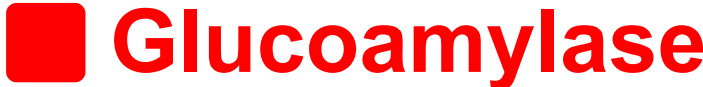

Supplement: Supplementary file 4 [file mmc4.pdf]
