# Supplementary material for: Expansion of maltose/sucrose related transporters in Ascomycetes and their association with corresponding disaccharide utilization
Source: Curr Res Microb Sci. 2025 Mar 3;8:100368. doi: 10.1016/j.crmicr.2025.100368 (PMC11930586; doi:10.1016/j.crmicr.2025.100368)

Supplementary Fig. S5. Phylogenetic classification of GH32 from 24 selected fungi in this study and previously experimentally characterized fungal GH32 genes related to sucrose hydrolysis. N and Y were used to indicate the absence and presence of a signal peptide, respectively. GenBank accession numbers were shown for experimentally characterized enzymes, while the JGI protein IDs were shown for other enzymes. Only the clade containing the characterized fungal invertase is highlight in orange. The details of protein sequences and annotation are listed in Supplementary Table S7.

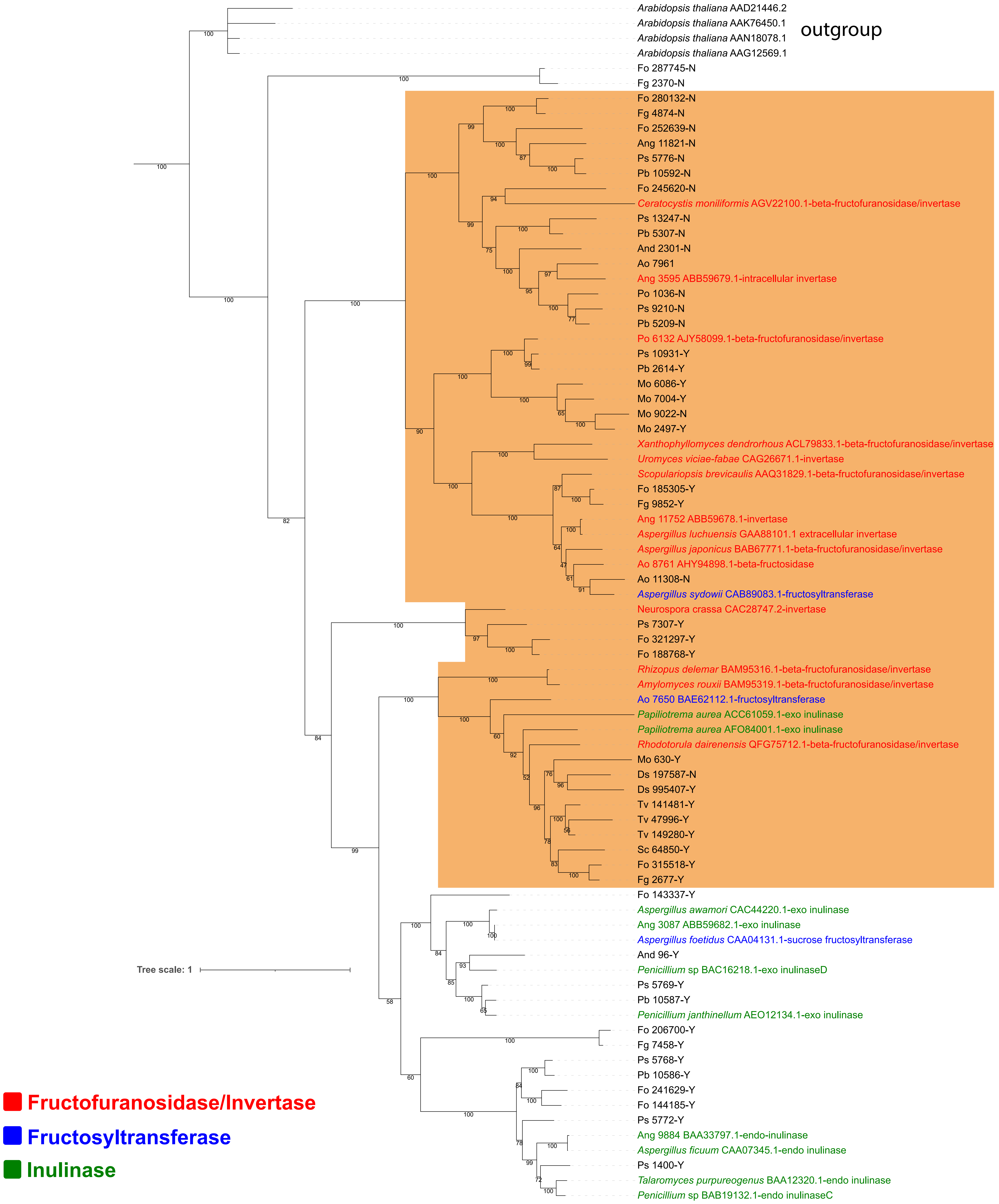

Supplement: Supplementary file 5 [file mmc5.pdf]
